# Spatiotemporal distribution of schistosomiasis transmission risk in Jiangling County, Hubei Province, P.R. China

**DOI:** 10.1371/journal.pntd.0011265

**Published:** 2023-05-04

**Authors:** Jiaxin Feng, Xia Zhang, Hehua Hu, Yanfeng Gong, Zhuowei Luo, Jingbo Xue, Chunli Cao, Jing Xu, Shizhu Li

**Affiliations:** 1 National Institute of Parasitic Diseases, Chinese Center for Disease Control and Prevention (Chinese Center for Tropical Diseases Research), NHC Key Laboratory of Parasite and Vector Biology, WHO Collaborating Centre for Tropical Diseases, National Center for International Research on Tropical Diseases, Shanghai, People’s Republic of China; 2 Jiangling Center for Disease Control and Prevention, Hubei province, People’s Republic of China; 3 The School of the Public Health of Fudan University, Shanghai, People’s Republic of China; 4 School of Global Health, Chinese Center for Tropical Diseases Research, Shanghai Jiao Tong University School of Medicine, Shanghai, People’s Republic of China; Baylor College of Medicine, UNITED STATES

## Abstract

**Objective:**

This study aims to explore the spatiotemporal distribution of schistosomiasis in Jiangling County, and provide insights into the precise schistosomiasis control.

**Methods:**

The descriptive epidemiological method and Joinpoint regression model were used to analyze the changes in infection rates of humans, livestock, snails, average density of living snails and occurrence rate of frames with snails in Jiangling County from 2005 to 2021. Spatial epidemiology methods were used to detect the spatiotemporal clustering of schistosomiasis transmission risk in Jiangling county.

**Results:**

The infection rates in humans, livestock, snails, average density of living snails and occurrence rate of frames with snails in Jiangling County decreased from 2005 to 2021 with statistically significant. The average density of living snails in Jiangling County was spatially clustered in each year, and the Moran’s *I* varied from 0.10 to 0.26. The hot spots were mainly concentrated in some villages of Xionghe Town, Baimasi Town and Shagang Town. The mean center of the distribution of average density of living snails in Jiangling County first moved from northwest to southeast, and then returned from southeast to northwest after 2014. SDE azimuth fluctuated in the range of 111.68°-124.42°. Kernal density analysis showed that the high and medium-high risk areas of Jiangling County from 2005 to 2021 were mainly concentrated in the central and eastern of Jiangling County, and the medium-low and low risk areas were mainly distributed in the periphery of Jiangling County.

**Conclusions:**

The epidemic situation of schistosomiasis decreased significantly in Jiangling County from 2005 to 2021, but the schistosomiasis transmission risk still had spatial clustering in some areas. After transmission interruption, targeted transmission risk intervention strategies can be adopted according to different types of schistosomiasis risk areas.

## Author summary

*Schistosoma* (*S*.) *japonicum* was once hyper-endemic in southern China. Jiangling County is one of the key endemic areas of schistosomiasis in Hubei Province, P.R. China. Although the prevalence of schistosomiasis in Jiangling County has decreased significantly, the geographical location of Jiangling County is suitable for snails breeding due to the crisscrossed ditches, so the risk of schistosomiasis transmission is still widespread. This study analyzed the schistosomiasis transmission risk in different regions in Jiangling County from 2005 to 2021, and targeted transmission risk intervention strategies can be adopted in different endemic areas after schistosomiasis transmission interruption. The areas with high risk of schistosomiasis transmission in Jiangling County were mainly distributed in the central part of Jiangling County near the west main ditch and the eastern part of Jiangling County. It is suggested strengthen schistosomiasis risk monitoring in Jiangling County, especially snails monitoring and snails elimination. In addition, health education intervention should be strengthened in key areas to prevent the spread of transmission risk to surrounding areas, in order to achieve the goal of eliminating schistosomiasis as soon as possible.

## 1. Introduction

Schistosomiasis remains an important public health issue across Africa, South America, and Asia [1]. The World Health Organization (WHO) estimates that schistosomiasis is transmitted in over 78 countries, and an estimated 779 million people are at risk of schistosomiasis [2,3]. Disability-adjusted life years (DALYs) of schistosomiasis is estimated as 3.3 million per year [4]. *Schistosoma* (*S*.) *japonicum* (Schistosomiasis for short) was once hyper-endemic in southern China [5]. Hubei Province is located at the middle reaches of the Yangtze River, affected by the east asian monsoon, it receives frequent precipitation and abundant water, and is one of the provinces with serious schistosomiasis endemic in China. In the early 1950s, there were more than 1.1 million schistosomiasis patients in the province, and the infection rate in humans was 28.39%, the highest infection rate in snails was 4.36%, and the infection rate in livestock was 21.40% [6].

Jiangling County is one of the key endemic areas of schistosomiasis in Hubei Province [7,8]. According to historical data, the number of schistosomiasis patients in the county reached 46,038 in 1970, including 359 acute schistosomiasis patients and 714 advanced schistosomiasis patients [8]. The infection rates of schistosomiasis in humans and livestock were 28.69% and 16.97%, respectively [8]. In 2004, the number of patients in the county still reached 20237, and the infection rates of schistosomiasis in humans and livestock were 10.22% and 7.59%, respectively, which had caused serious harm to people’s health in epidemic areas [9]. In 2004, Jiangling County implemented a schistosomiasis comprehensive prevention and control strategy based on infectious source control, and achieved remarkable effectiveness [10]. By 2014, the infection rate of schistosomiasis in humans in Jiangling County decreased from 11.83% in 2004 to 0 [10]. Although the endemic situation of schistosomiasis in Jiangling County has decreased significantly, the geographical location of Jiangling County is suitable for snails breeding due to the complex water system and crisscrossed ditches, and the epidemic factors affecting the transmission of schistosomiasis have not been completely changed, so the risk of schistosomiasis transmission is still widespread [11,12]. Therefore, it is of great significance to explore the spatial and temporal distribution characteristics of schistosomiasis transmission risk in Jiangling County, and to identify the transmission risk of schistosomiasis in different areas.

The Joinpoint regression model is a specialized statistical model for analyzing temporal trends in morbidity or mortality [13]. In recent years, this model has been widely used in the fields of mortality change of malignant tumors and epidemiology of chronic diseases, but it is rarely used in schistosomiasis-related research [14,15]. With the rapid development of geographic information system (GIS) and spatial analysis technology, spatial epidemiology has been widely used in the field of infectious disease research [16–20]. Spatial epidemiology can describe, quantify and explain the spatial distribution characteristics and changes of diseases, and provide suggestions for disease prevention, health promotion and health resource allocation [21,22]. For example, Li *et al*. [23] used spatiotemporal clustering analysis to explore the effectiveness of a new integrated control strategy that was implemented by the national control program since 2004. Gong *et al*. [24] analyzed the spatiotemporal clustering of schistosomiasis in China from 2005 to 2019. Based on the Joinpoint regression model to analyze the schistosomiasis epidemic trend in Jiangling County from 2005 to 2021, the spatial epidemiological analysis method was used to explore the spatiotemporal clustering characteristics of schistosomiasis transmission risk in Jiangling County from 2005 to 2021 in this study, to provide insights into the schistosomiasis transmission risk intervention in Jiangling County after transmission interruption.

## 2. Methods

### 2.1. Ethics statement

This study was approved by the Ethics Review Committee of National Institute of Parasitic Diseases, Chinese Center for Disease Control and Prevention (National Center for Tropical Diseases Research) (Ethics Approval number: 2021019). The study involved collecting blood and feces from residents with the written consent of all respondents or their parents. The interests of the people involved have been fully protected, and the possible benefits of the subjects outweigh the possible risks.

### 2.2. Study site

**Fig 1:**
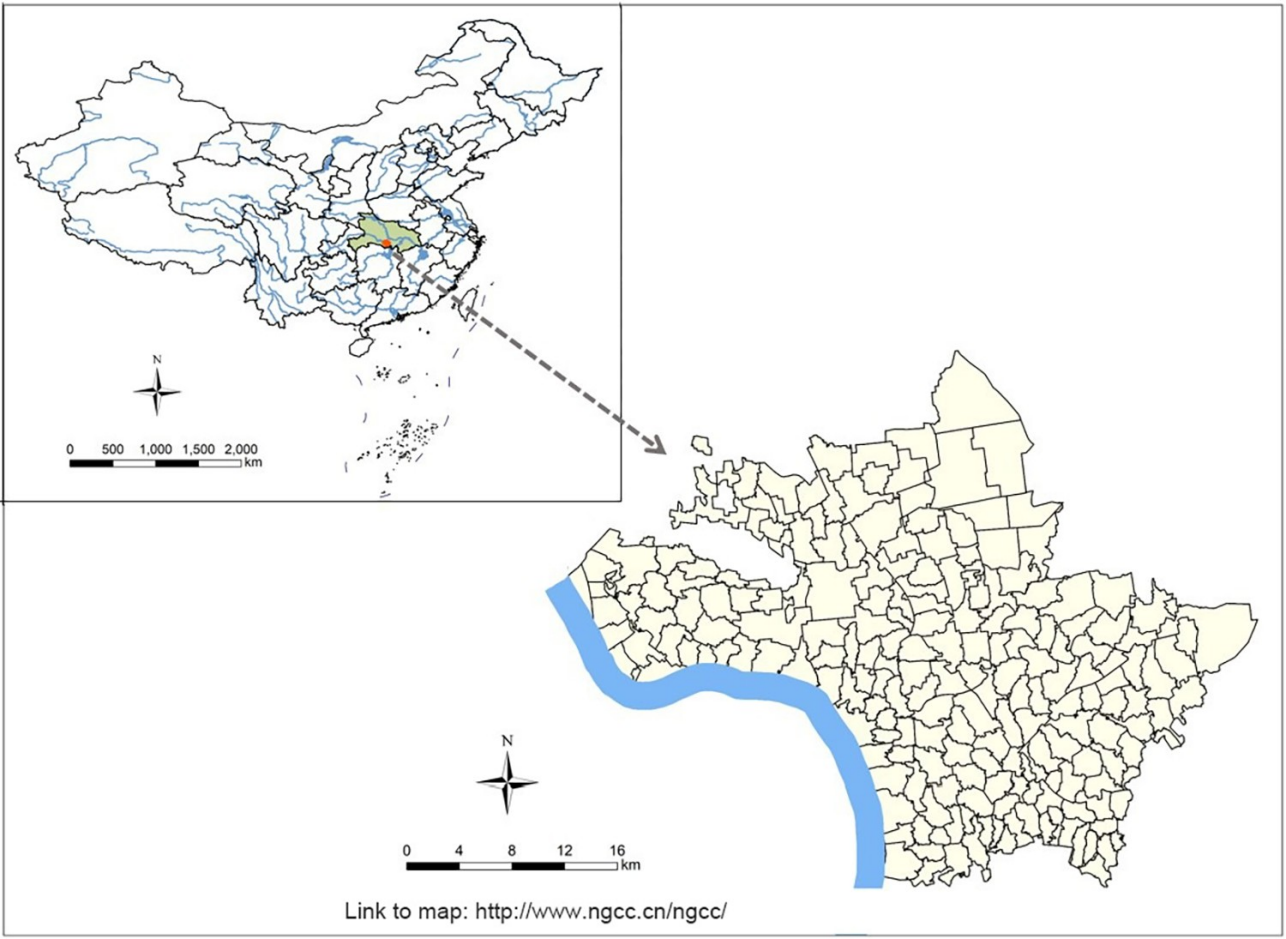
The map of the study site in Jiangling County, Hubei Province, People’s Republic of China. The base layer of the figure in paper is from http://www.ngcc.cn/ngcc/ with credit to National Geomatics Center of China.

Jiangling County is located at the central and southern part of Hubei Province, on the north bank of the Jingjiang River section in the middle reaches of the Yangtze River, and was once a serious endemic area of schistosomiasis in Hubei Province [7,8] (Fig 1). Jiangling County is known as a typical lake and marshland endemic area of schistosomiasis with unique geographical environment and climate, numerous lakes, crisscrossed ditches, dense vegetation and frequent precipitation, which are very suitable for snails breeding. The county has 11 towns (administrative areas and farms) and 198 administrative villages (subfields and teams), all of which belong to schistosomiasis endemic areas, and is the key endemic county for schistosomiasis prevention and control in Hubei Province.

### 2.3. Data collection

The data on schistosomiasis in humans, livestock and investigation on snails in Jiangling County from 2005 to 2021 were collected. Permanent residents aged 6 to 65 years underwent serological screening (IHA) for schistosomiasis infection, as well as measurements of antibody titers. Seropositive individuals were tested for parasites by the Kato-Katz technique and miracidium hatching test [9,25]. The hatching test was used to detect schistosomiasis infections in livestock. Snails investigation was carried out by systematic sampling combined with environmental sampling in environments with snails presence and suspected snails presence, the size of a frame is 33cm×33cm, and line spacing and frame spacing are both 20 meters, global position system (GPS) is used to locate environments where snails presence [9,25]. The captured snails were observed by crushing microscopic examination to identify the dead or alive and infected snails.

### 2.4. Epidemic indicators calculation

The schistosomiasis epidemic data of Jiangling County from 2005 to 2021 were collected by administrative villages. The schistosomiasis infection rates in humans, livestock and snails, the average density of living snails and the occurrence rate of frames with snails were calculated.

The formulas are calculated as follows:

$$Infection\;rate\;in\;humans(\%)=\frac{the\;number\;of\;positive\;serological\;tests}{the\;number\;of\;serological\;tests}\times \frac{the\;number\;of\;positive\;pathogenic\;tests}{the\;number\;of\;pathogenic\;tests}\times 100\%$$

$$Infection\;rate\;in\;livestock(\%)=\frac{the\;number\;of\;livestock\;pathogenic\;tests\;positive}{the\;number\;of\;livestock\;tests}\times 100\%$$

$$Infection\;rate\;in\;snails(\%)=\frac{the\;number\;of\;infectious\;snails\;detected}{the\;number\;of\;live\;snails}\times 100\%$$

$$Average\;density\;of\;living\;snails(piece/frame)=\frac{the\;number\;of\;live\;snails}{the\;number\;of\;frames}$$

$$Occurrence\;rate\;of\;frames\;with\;snails(\%)=\frac{the\;number\;of\;frames\;with\;snails}{the\;number\;of\;frames}\times 100\%$$

### 2.5. Data analysis

#### 2.5.1. Establishment of spatial database of epidemic situation

The zoning map of Jiangling County was extracted from the national county boundary electronic map, and the hand-held GPS instrument was used to locate the village committee of each administrative village. In ArcGIS software, version 10.7, the village-level epidemic data were associated with the vectoring electronic map of Jiangling County with the name of administrative village as the association field, and the spatial analysis database of schistosomiasis epidemic situation in Jiangling County was generated. WGS_1984 was used as the geographic coordinate system and WGS_1984_UTM_Zone_49N was used as the projection coordinate system.

#### 2.5.2. Joinpoint model temporal trend analysis

The epidemic data of schistosomiasis in Jiangling County from 2005 to 2021 were loaded into Microsoft Excel 2019 to establish a database, and the Joinpoint Regression Program software (version 4.8.0.1) was used for modeling to analyze the changes in schistosomiasis infection rates in humans, livestock and snails, the average density of living snails and the occurrence rate of frames with snails in Jiangling County. The annual percent change (APC) and average annual percent change (AAPC) of each epidemic indicator were calculated [14]. The formula is calculated as follows:

$$APC/AAPC=(e^{\beta }-1)\times 100\%$$

*β* is the regression coefficient, *e* is the error, and *T*-tests are used for APC tests. If APC = AAPC, there is no joinpoint, indicating that the data generally shows a monotonically increasing or decreasing trend. The Joinpoint model used mathematical algorithms to judge whether there was a significant change in the schistosomiasis trend in a certain period of time [26–28], and *P*<0.05 was considered statistically significant. Since the model does not support data with a percentage of zero, the observation with a percentage of zero was replaced by the minuscule data 0.00001 in the analysis.

#### 2.5.3. Spatial autocorrelation analysis

Spatial autocorrelation analysis is a spatial analysis method that studies whether the observed values of a variable in a certain location are correlated with the observed values of the same variable in adjacent locations and the degree of correlation [29,30]. ArcGIS software (version 10.7) was used for spatial autocorrelation analysis in this study. Global Moran’s *I* statistic can describe the overall spatial distribution characteristics of the study area. By calculating the global spatial autocorrelation statistic Moran’s *I*, we can analyze whether there is a spatial correlation in the spatial distribution of the average density of living snails and calculate the degree of correlation. The value of global Moran’s *I* statistic ranges from -1 (perfect dispersion) to 1 (perfect correlation). Negative values indicate negative spatial autocorrelation, positive values indicate positive spatial autocorrelation, and a value of zero value indicates a random spatial pattern (no spatial correlation) [31–33]. Anselin Local Moran’s *I* can judge the specific location and spatial distribution type of spatially related regions based on global analysis. After normalization, the regions can be divided into five different types: (1) high-high; (2) low-low; (3) high-low; (4) low-high; and (5) no significant local spatial autocorrelation [34].

#### 2.5.4. Hot spots analysis

The “Getis-Ord Gi*” tool in ArcGIS software version 10.7 was used to analyze the average density of living snails in Jiangling County from 2005 to 2021. Hot spots analysis can calculate the Getis-Ord Gi* statistic for each element in the dataset, and the obtained Z-score and *P*-value can further identify the locations where statistically significant high (hot spots) or low (cold spots) values of the average density of living snails are spatially clustered [35,36].

#### 2.5.5. Central tendency and dispersion analysis

The “mean center” and “direction distribution tool” in ArcGIS software version 10.7 were used to explore the spatial dispersal direction and change trend of the average density of living snails in Jiangling County from 2005 to 2021. The data from 2005 to 2021 were divided into one group every three years. The mean center, also known as the spatial mean, was used as the central tendency indicator, which refers to the center or average position of a group of points. The direction deviation was described by standard deviation ellipse (SDE), also known as the direction distribution tool. The long and short axes of the standard deviation ellipse represent the spatial diffusion direction of the average density of living snails, and the size of the ellipse represents the clustering range of the average density of living snails in the region and time[37].

#### 2.5.6. Kernal density analysis

Kernal density analysis of point elements was used to calculate the density of point elements around each output raster pixel [38]. In ArcGIS software version 10.7, the parameter “Population” was set as the average density of living snails to assign weights. The results of the kernal density analysis were divided into 5 levels by the “Natural Breaks” method. According to the range of the value from low to high, they were respectively regarded as low risk area, medium low risk area, medium risk area, medium high risk area and high risk area.

## 3. Results

### 3.1. Trends of the schistosomiasis epidemic

**Fig 2:**
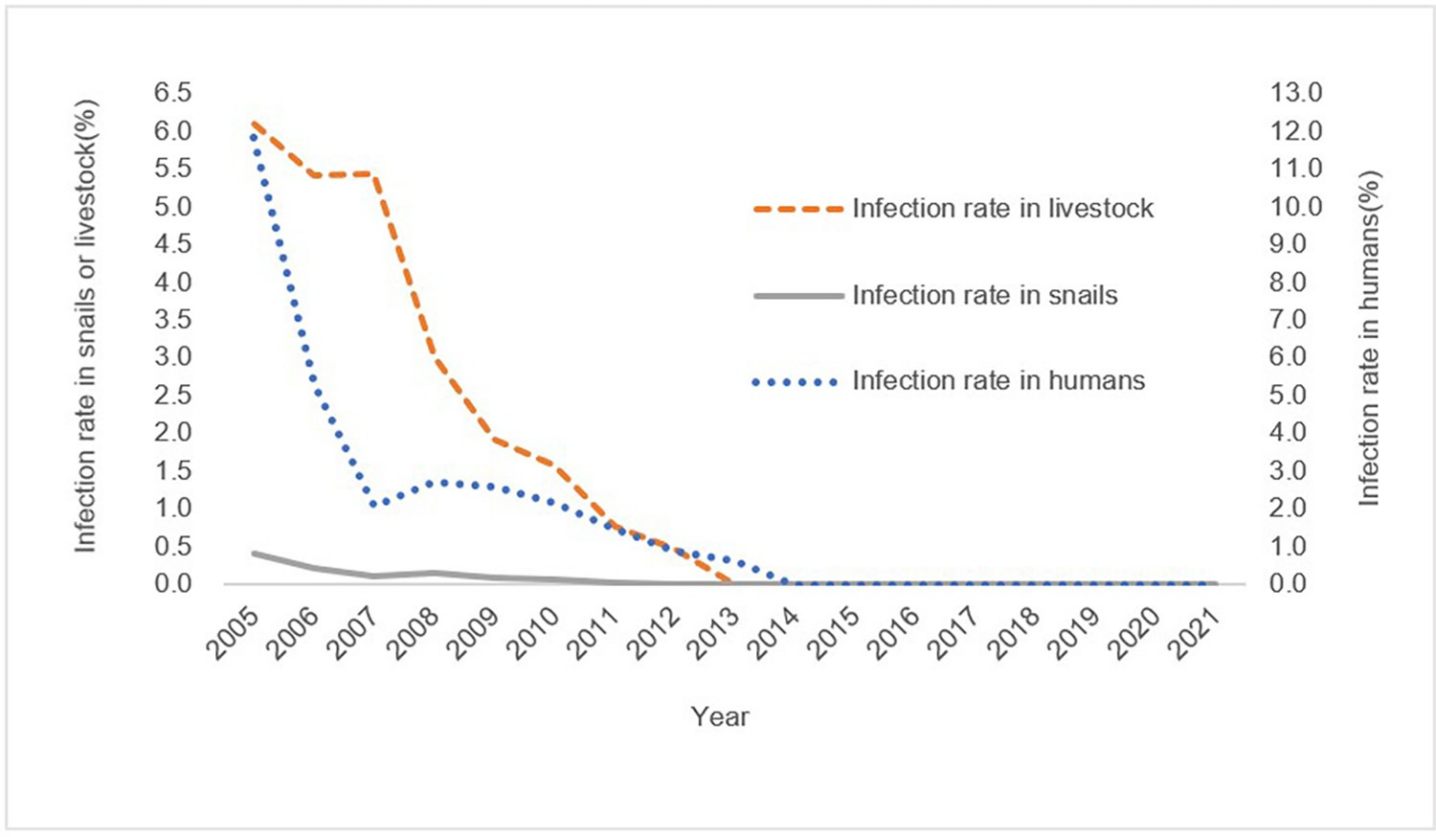
Changes in the prevalence of schistosomiasis infection in humans, livestock, and snails in Jiangling County from 2005 to 2021.

**Fig 3:**
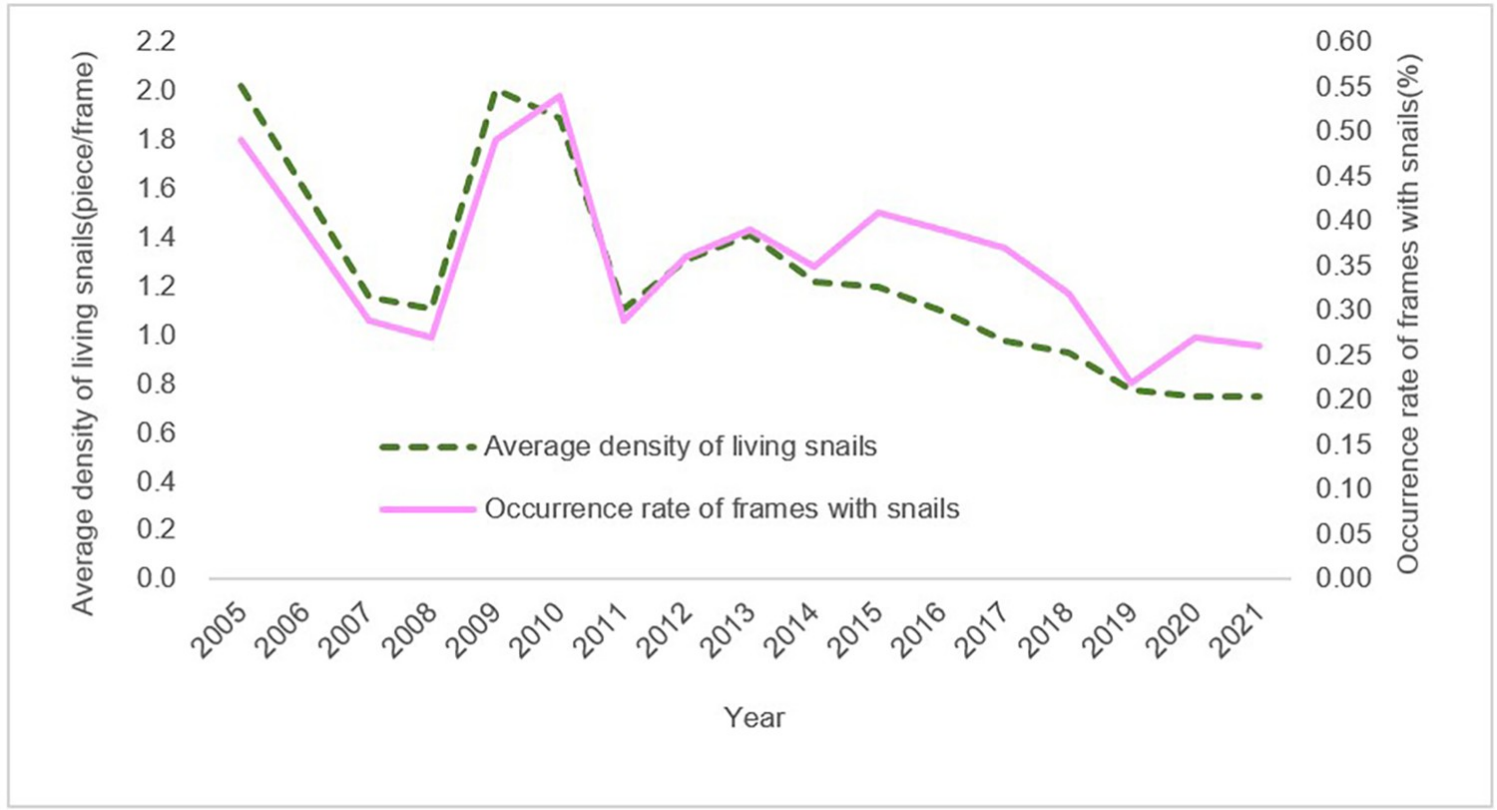
Changes in the average density of living snails and the occurrence rate of frames with snails in Jiangling County from 2005 to 2021.

The prevalence of schistosomiasis infection trended toward a continuous decline in humans, livestock, and snails in Jiangling County from 2005 to 2021 (Table 1). The infection rate in humans decreased from 11.83% in 2005 to 0 in 2021, the infection rate in livestock decreased from 6.11% in 2005 to 0 in 2021, and the infection rate in snails decreased from 0.41% in 2005 to 0 in 2021 (Fig 2). The average density of living snails and the occurrence rate of frames with snails in Jiangling County from 2005 to 2021 had a slight fluctuation from 2009 to 2011, and then showed a downward trend as a whole. The average density of living snails in Jiangling County decreased from 2.02% in 2005 to 0.75% in 2021, a decrease of 62.87%. The occurrence rate of frames with snails in Jiangling County decreased from 0.49% in 2005 to 0.26% in 2021, a decrease of 46.94% (Fig 3). There have been no cases of acute schistosomiasis in the county since 2008, and no infected snails have been found since 2012.

**Table 1:** Investigation results of schistosomiasis in humans, livestock and investigation on snails in Jiangling County from 2005 to 2021.

	No. of people in endemic village	No. of people with serum tests	No. of people with positive serum test	No. of people with stool tests	No. of people with positive stool test	No. of livestock in stock	No. of livestock with stool tests	No. of livestock with positive stool test	No. snail environment surveyed	No. frames surveyed of snail	No. frames with snail	No. living snails
2005	298618	100014	35541	12024	3999	15509	15150	926	9358	524071	256681	1060309
2006	298618	98808	17148	15067	4564	15365	14150	1640	11945	668947	260889	1063625
2007	297437	171804	22630	22280	3619	15484	12000	654	14441	813824	235580	945461
2008	297503	89141	14207	14157	2553	10239	10239	308	13650	763380	206468	848044
2009	299051	87316	13086	12994	2158	10239	10239	196	6620	437876	216630	878387
2010	299150	75579	11264	11021	1602	11732	11732	185	6606	526754	255545	997641
2011	299150	83264	9187	8942	1199	2470	2470	19	12487	953536	279882	1054646
2012	274011	69638	6988	6711	573	3468	3468	16	7902	633218	228444	831835
2013	274181	65019	4682	4518	395	0	0	0	5975	617544	241010	869860
2014	109766	52175	4455	4343	229	0	0	0	7484	647778	225436	791592
2015	109766	58219	3365	3248	74	0	0	0	8608	420686	163539	504877
2016	97733	46848	4003	3916	0	0	0	0	7690	740050	292034	812443
2017	88990	57621	2852	2852	0	0	0	0	7411	720100	266088	704475
2018	104954	49436	2330	2330	0	0	0	0	5145	169752	55090	158398
2019	134974	41081	2310	2310	0	0	0	0	4992	224343	48424	175478
2020	126606	41785	1417	1417	0	0	0	0	5233	229481	61641	171445
2021	114019	37739	680	680	0	0	0	0	5149	201244	52251	151870

**Fig 4:**
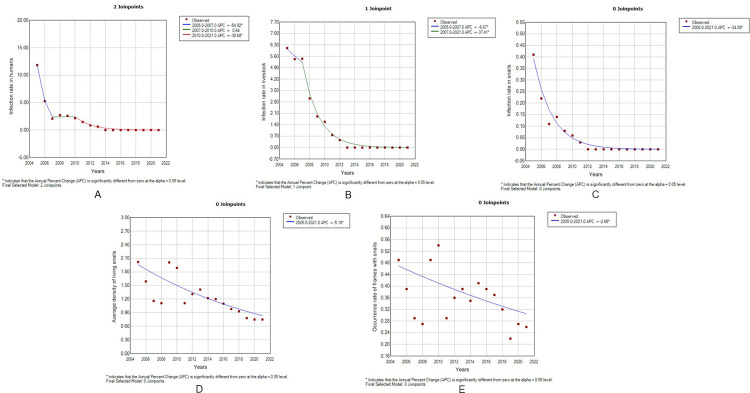
Pictures of joinpoint model analysis on trends of schistosomiasis-related epidemic indicators in Jiangling County from 2005 to 2021 (A: infection rate in humans; B: infection rate in livestock; C: infection rate in snails; D: average density of living snails; E:occurrence rate of frames with snails).

Joinpoint model analysis showed that the infection rate in humans in Jiangling County decreased at the fastest rate from 2005 to 2021 (AAPC = -35.2%, *P* < 0.05), with two joinpoints, and the period from 2005 to 2021 was divided into three periods. Except for the period from 2007 to 2010, the decline rates of the other two periods were statistically significant. The infection rate in livestock in Jiangling County from 2005 to 2021 showed one joinpoint (AAPC = -34.2%, *P*<0.05). The period from 2005 to 2021 was divided into two periods, and the decline rates in the two periods were statistically significant. There were statistically significant decreasing rates of infection rate in snails, average density of living snails and occurrence rate of frames with snails in Jiangling County from 2005 to 2021 (APC = AAPC = -34.1%, -5.2%, -2.7%, *P*<0.05), but no significant joinpoint was found in the decreasing trend (Table 2 and Fig 4).

**Table 2:** Results of Joinpoint model analysis on trends of schistosomiasis-related epidemic indicators in Jiangling County from 2005 to 2021.

Indicators	Time period	*t* value	*P* value	APC/AAPC	Lower limit of 95% CI	Upper limit of 95% CI
Infection rate in humans	2005–2007	-143.8	<0.05	-54.9	-55.5	-54.4
	2007–2010	0.4	0.7	0.6	-2.5	3.9
	2010–2021	-23.3	<0.05	-38.7	-41.5	-35.7
	2005–2021	-29.6	<0.05	-35.2	-37.1	-33.4
Infection rate in livestock	2005–2007	-3.0	<0.05	-6.7	-11.2	-1.9
	2007–2021	-19.2	<0.05	-37.4	-40.7	-34.0
	2005–2021	-19.4	<0.05	-34.2	-36.9	-31.4
Infection rate in snails	2005–2021	-13.9	<0.05	-34.1	-38.2	-29.7
Average density of living snails	2005–2021	-4.7	<0.05	-5.2	-7.5	-2.9
Occurrence rate of frames with snails	2005–2021	-2.4	<0.05	-2.7	-4.9	-0.3

### 3.2. Spatial autocorrelation analysis

The global Moran’s *I* spatial autocorrelation analysis of the average density of living snails in Jiangling County from 2005 to 2021 showed that the global Moran’s *I* statistic >0 in each year, and the variation range was 0.10–0.26. Monte-Carlo test showed that the global Moran’s *I* statistic of each year was statistically significant at the test level of *P*<0.01, indicating that the average density of living snails in Jiangling County from 2005 to 2021 was not randomly distributed in the whole county, with a positive spatial correlation, suggesting that the average density of living snails in Jiangling County was spatially clustered. The degree of clustering was the highest in 2013 (Moran’s *I* = 0.26) and the lowest in 2005 (Moran’s *I* = 0.10) (Table 3).

**Table 3:** Global Moran’s *I* spatial autocorrelation analysis on average density of living snails in Jiangling County from 2005 to 2021.

Year	Moran’s *I* statistic	*Z* score	*P* value	Clustered
2005	0.10	4.22	<0.01	Yes
2006	0.13	5.01	<0.01	Yes
2007	0.14	5.45	<0.01	Yes
2008	0.14	5.53	<0.01	Yes
2009	0.21	8.26	<0.01	Yes
2010	0.22	8.68	<0.01	Yes
2011	0.14	5.72	<0.01	Yes
2012	0.21	8.47	<0.01	Yes
2013	0.26	8.92	<0.01	Yes
2014	0.24	9.39	<0.01	Yes
2015	0.21	8.41	<0.01	Yes
2016	0.21	8.46	<0.01	Yes
2017	0.24	9.47	<0.01	Yes
2018	0.17	6.90	<0.01	Yes
2019	0.13	5.32	<0.01	Yes
2020	0.12	5.01	<0.01	Yes
2021	0.14	5.77	<0.01	Yes

**Fig 5:**
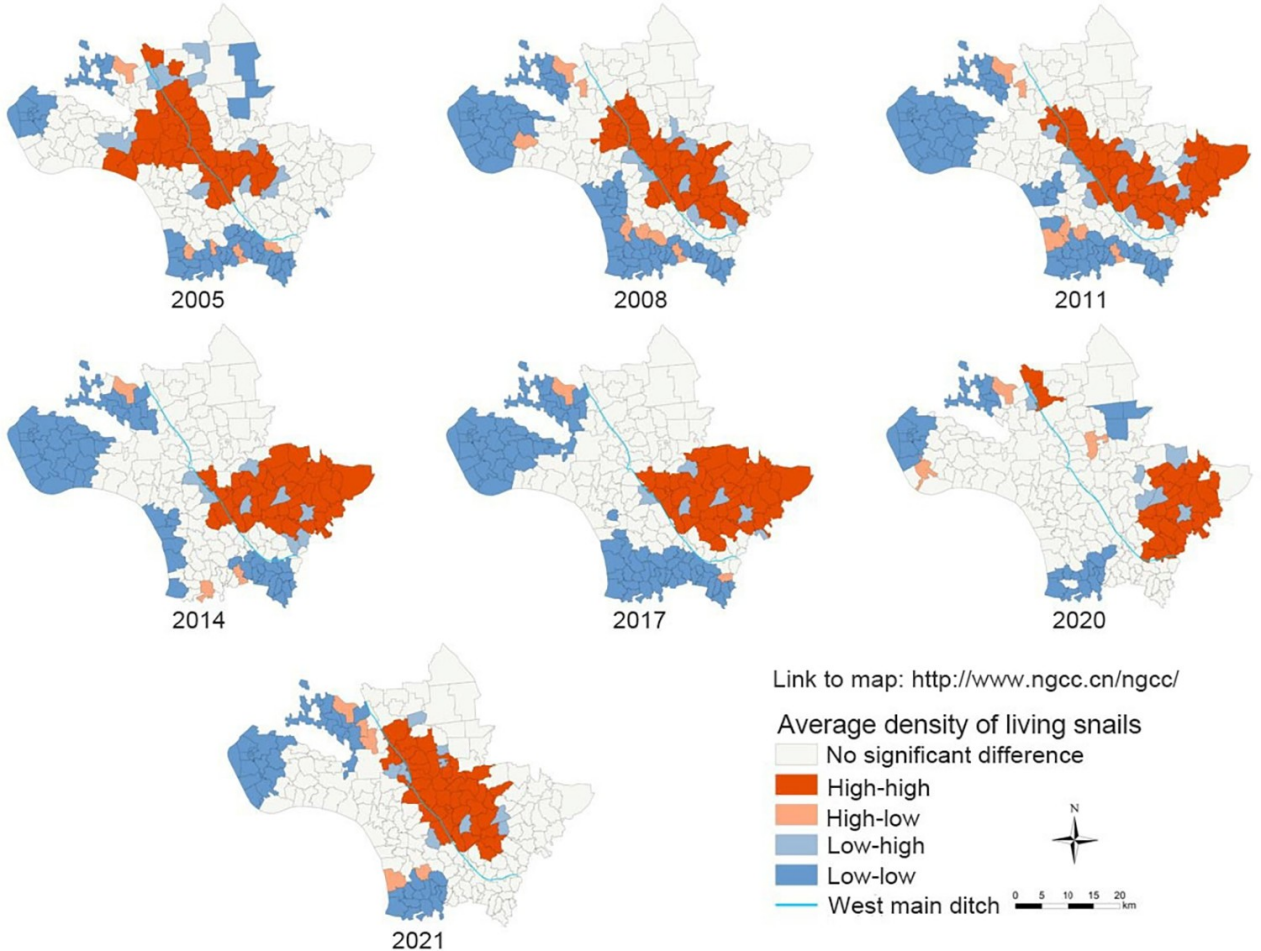
Local spatial autocorrelation analysis of the average density of living snails in Jiangling County from 2005 to 2021. The base layer of the figure in paper is from http://www.ngcc.cn/ngcc/ with credit to National Geomatics Center of China.

To further explore the specific clustering areas of the average density of living snails in Jiangling County from 2005 to 2021, local Moran’s *I* statistic was conducted to analyze the average density of living snails. The results showed that the average density of living snails in Jiangling County from 2005 to 2021 showed five types of clustering areas. The “high-high” clustering areas were mainly located near the west main ditch in the middle of Jiangling County and in the eastern part of Jiangling County. Around 2005, it was mainly distributed in the north of Xionghe Town and the southwest of Baimasi Town. Since 2008, “high-high” clustering areas have also been detected in Shagang Town. Around 2021, the “high-high” clustering areas again concentrated in Baimasi Town. There was little change in the “low-low” clustering areas, mainly in the western and southern areas of Jiangling County, mainly in Majiazhai Town and Puji Town. The “low-high” clustering areas were mainly around the “high-high” clustering areas, and their locations were distributed everywhere (Fig 5).

### 3.3. Hot spots analysis

**Fig 6:**
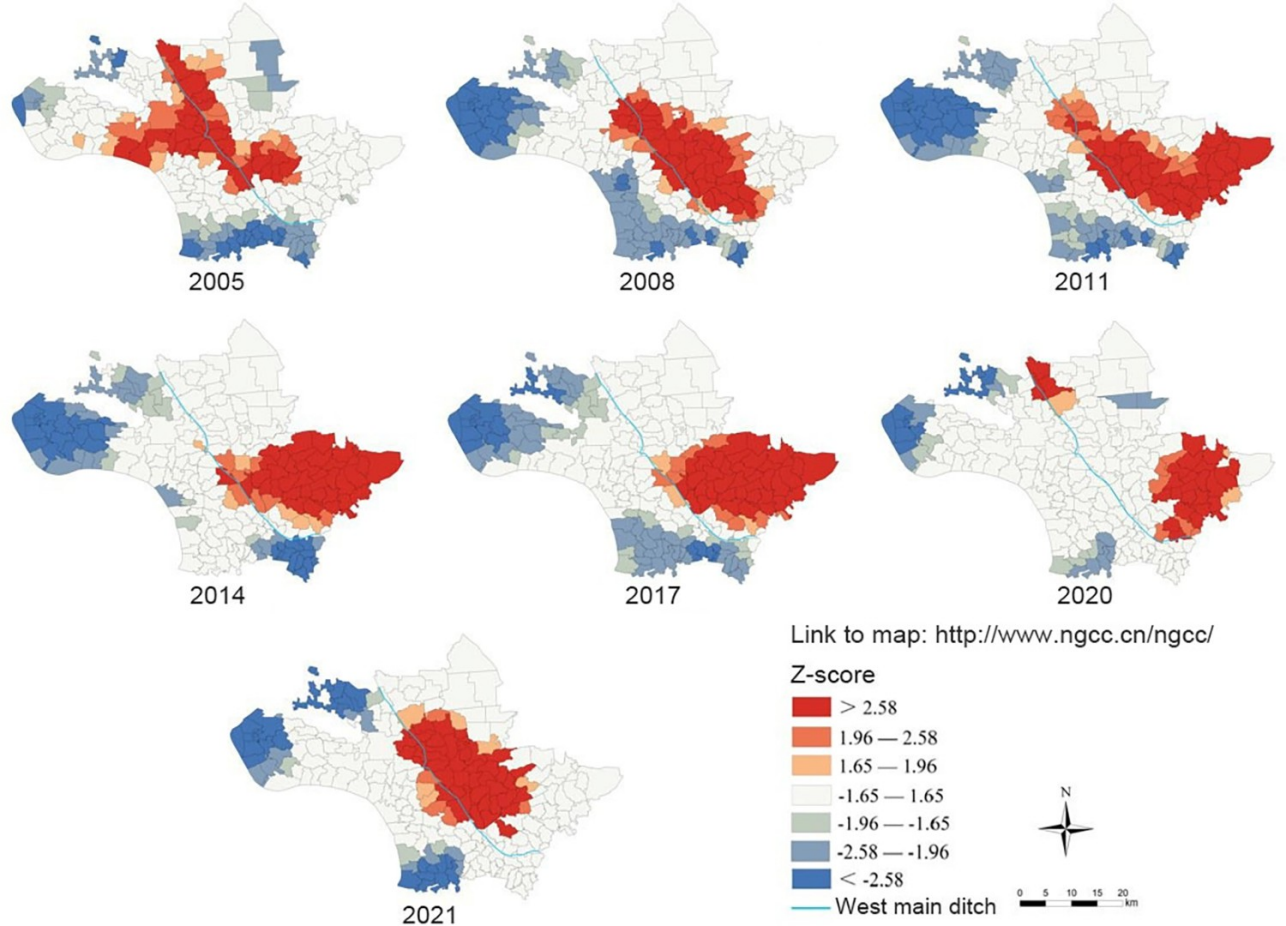
Distribution of hot spot areas for the average density of living snails in Jiangling County from 2005 to 2021. The base layer of the figure in paper is from http://www.ngcc.cn/ngcc/ with credit to National Geomatics Center of China.

The hot spots analysis results showed that the hot spots areas of the average density of living snails in Jiangling County from 2005 to 2021 were mainly concentrated in the central and eastern areas of Jiangling County. The hot spots clustering areas of the average density of living snails in Jiangling County from 2005 to 2011 were large, mainly distributed along the west main ditch, and the hot spots clustering areas decreased slightly from 2014 to 2021 (Fig 6).

### 3.4. Central tendency and dispersion analysis

**Fig 7:**
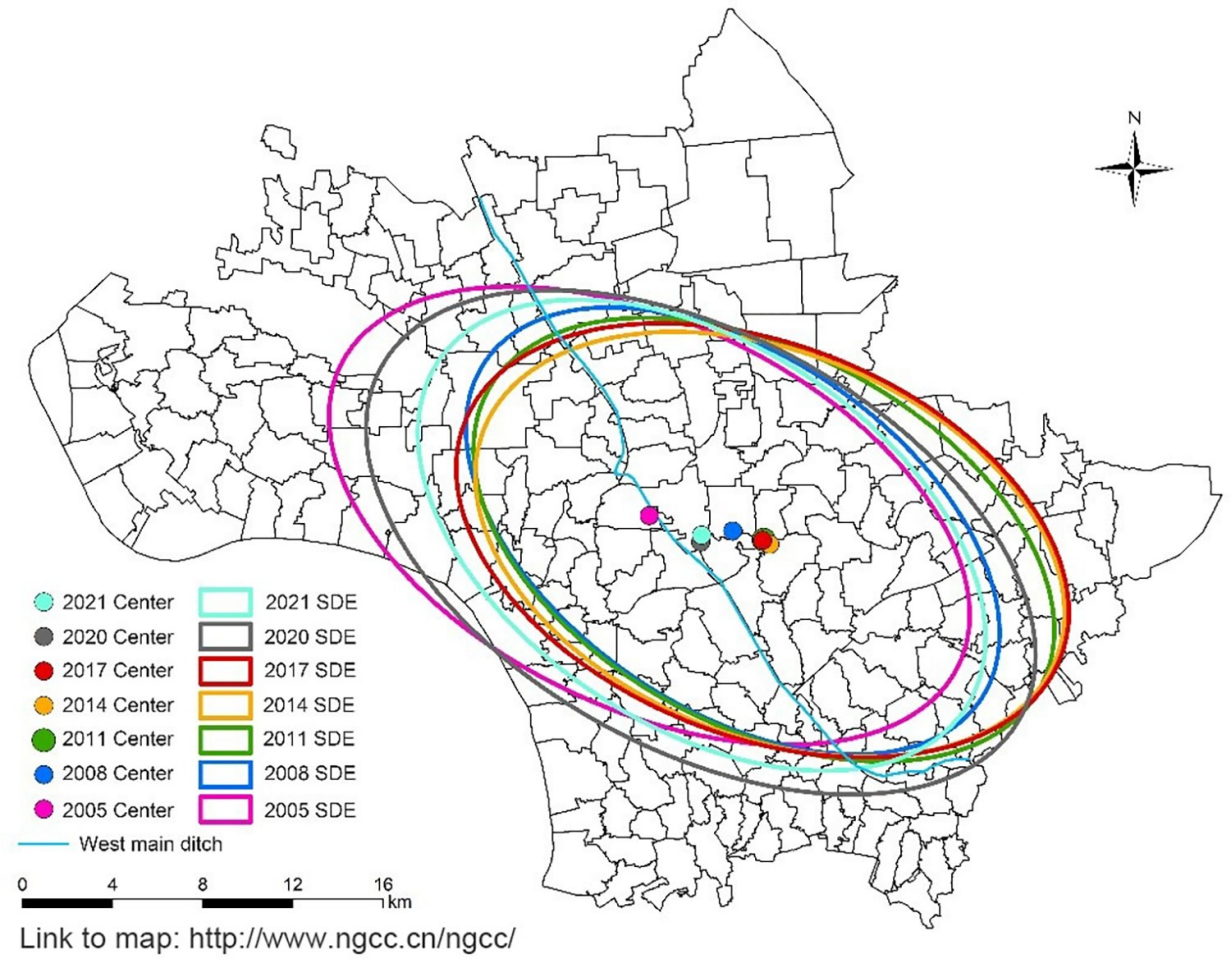
Central and discrete trend changes for the average density of living snails in Jiangling County from 2005 to 2021. The base layer of the figure in paper is from http://www.ngcc.cn/ngcc/ with credit to National Geomatics Center of China.

Fig 7 shows the mean center and standard deviation ellipse changes in the distribution of the average density of living snails in Jiangling County from 2005 to 2021. The mean center of the distribution of the average density of living snails in Jiangling County first moved from northwest to southeast, and then returned from southeast to northwest after 2014, mainly distributed in the areas along the west main ditch in the east of Xionghe Town and the west of Baimasi Town. The SDE azimuth of the distribution of the average density of living snails in Jiangling County fluctuated in the range of 111.68°-124.42°, indicating that the distribution of the average density of living snails in Jiangling County had a clear direction, and the overall direction was from southeast to northwest.

### 3.5. Kernal density analysis

**Fig 8:**
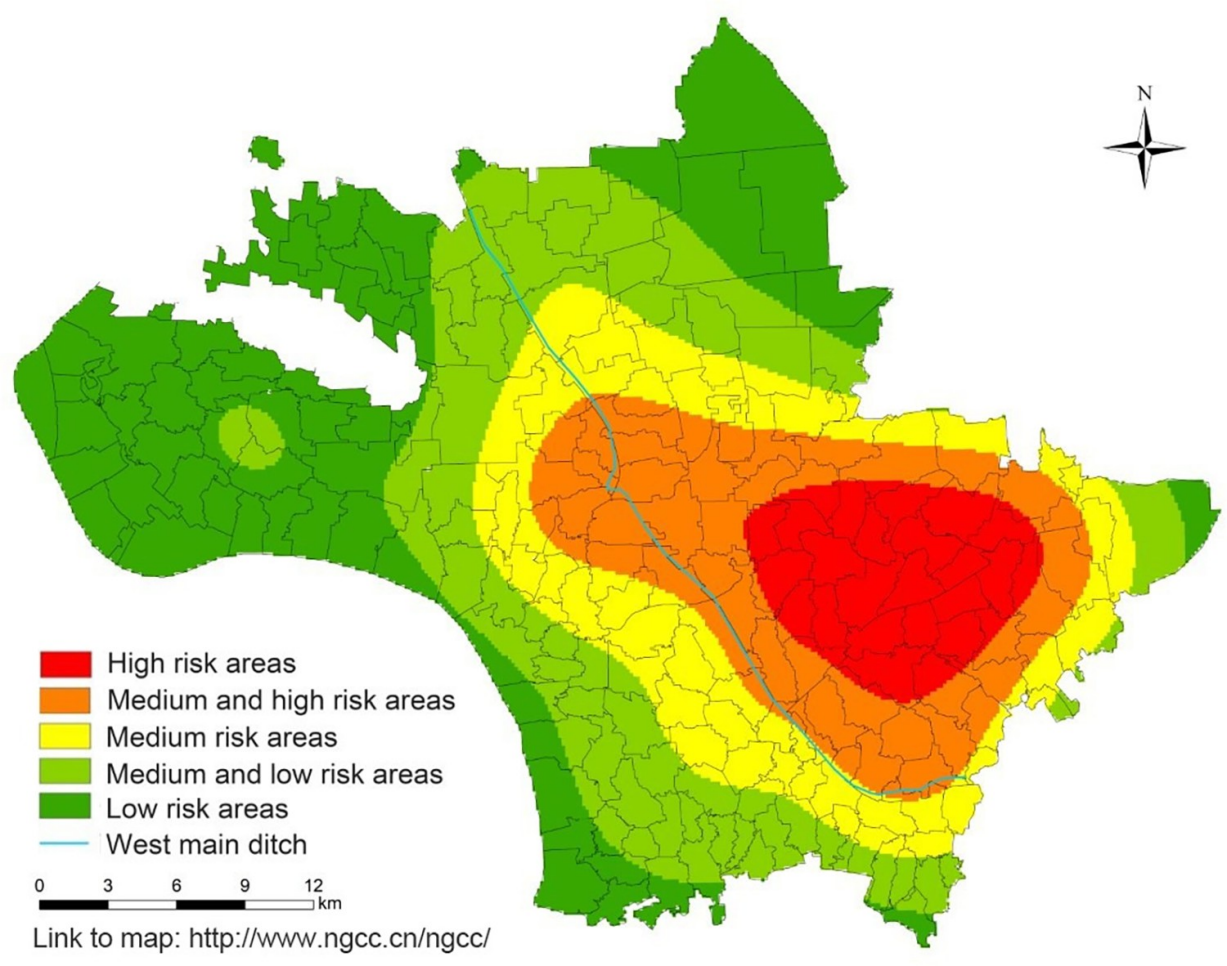
Analysis of kernal density for the average density of living snails in Jiangling County from 2005 to 2021. The base layer of the figure in paper is from http://www.ngcc.cn/ngcc/ with credit to National Geomatics Center of China.

The overall transmission risk distribution of Jiangling County from 2005 to 2021 was detected by kernal density analysis. The results showed that there was one high risk area in Jiangling County from 2005 to 2021, and each risk area was spread out in an oval shape with the high risk area as the center. The high risk areas were mainly concentrated in the east of Baimasi Town and the west of Shagang Town, while the medium and high risk areas were distributed in the periphery of the high risk area, mainly concentrated in the southeast of Baimasi Town, the east of Xionghe Town, the middle of Shagang Town and the north of Qinshi Town. The medium-low and low risk areas are mainly distributed in the periphery of Jiangling County, mainly in Zishi Town, Majiazhai Town, Haoxue Town, Liuheyuan management area, Sanhu management area, Jiangbei farm, central and southern part of Puji Town and southern part of Qinshi Town (Fig 8).

## 4. Discussion

Since the founding of the People’s Republic of China, China has attached great importance to schistosomiasis control. The comprehensive schistosomiasis control strategies of “snails elimination”, “humans and livestock simultaneous chemotherapy” and “infection source control” have been implemented successively [39]. In 2004, schistosomiasis was listed as one of the four major infectious diseases for priority control, and in 2014, schistosomiasis was listed as one of the five major diseases for priority control [40,41]. After more than 70 years of active control, the epidemic situation of schistosomiasis in China has reached the lowest level in history, and the control progress is moving towards the elimination stage. Jiangling County is known as a typical lake and marshland endemic area of schistosomiasis in China, and used to be one of the key endemic areas. Since 2004, according to the requirements of the medium and long term program outline for schistosomiasis prevention and control in Hubei Province (2005–2015), Jiangling County has carried out comprehensive control focusing on the source of infection control, such as detection and treatment of schistosomiasis in humans and livestock, snails elimination, treatment of advanced schistosomiasis patients, and health education, which has effectively controlled the epidemic situation of schistosomiasis. By the end of 2008, the infection rate of schistosomiasis in humans in Jiangling County decreased from 11.83% in 2005 to 2.73%, and the whole county reached the control standard of schistosomiasis epidemic situation [10]. In 2009, the project of “joint action for schistosomiasis control by the province and the ministry” was launched. In 2010, in the mode of county-wide promotion and comprehensive control, the project of “replacing cattle with machines” was carried out to promote the construction of a “no-cattle county” [42]. In 2013, the infection rate of schistosomiasis in humans in Jiangling County decreased to 0.63%, and the whole county reached the standard of schistosomiasis transmission control. In 2017, the county met the standard for schistosomiasis transmission interruption.

The analysis of the schistosomiasis epidemic trend in Jiangling County from 2005 to 2021 showed that, after comprehensive control, the schistosomiasis epidemic situation in Jiangling County showed an overall downward trend since 2005. By 2021, the infection rates of schistosomiasis in humans, livestock and snails in Jiangling County went from 11.83%, 6.11% and 0.41% to 0, respectively. The average density of living snails and the occurrence rate of frames with snails decreased to less than 1%, and the decrease was about 50%. Joinpoint model analysis showed that the declining trend of other epidemic indicators was statistically significant (*P*<0.05), except for the infection rate in humans, which had no statistical significance from 2007 to 2010. The main reason may be that to achieve the goal of schistosomiasis epidemic control in 2008, Jiangling County implemented comprehensive chemotherapy and intensified the comprehensive schistosomiasis control measures focusing on the control of infection source, leading to a low level of infection rate in humans in 2007. However, after the standard of schistosomiasis epidemic control was reached in 2008, the awareness of schistosomiasis control was slightly relaxed so that the infection rate declined slowly. In 2010, after the implementation of “replacing cattle with machines”, the infection rate continued to decline rapidly. Liu *et al*.[9] analyzed the prevalence of schistosomiasis in Jiangling County from 2004 to 2013, and showed that the infection rate of schistosomiasis in human decreased rapidly from 2004 to 2008, and that of livestock decreased rapidly from 2009 to 2013. This study is consistent with their findings. In addition, the results of the Joinpoint model showed that the overall decreasing rates of schistosomiasis infection rates in humans, livestock, snails, the average density of living snails, and occurrence rate of frames with snails in Jiangling County from 2005 to 2021 were statistically significant, and there were several statistically significant joinpoints of schistosomiasis infection rates in humans and livestock. The results indicated that schistosomiasis control in Jiangling County had achieved remarkable results and the epidemic situation of schistosomiasis had been effectively controlled. However, due to the large variety and number of schistosomiasis infection sources, wide distribution of intermediate host snails, and insufficient sensitivity of existing detection techniques, the results of schistosomiasis control are still fragile, and the risk of schistosomiasis transmission will exist in a certain period and scope for a long time [12]. Therefore, it is still necessary to explore the spatial and temporal distribution characteristics of schistosomiasis in Jiangling County, identify the transmission risk of schistosomiasis in different areas of Jiangling County, to take targeted transmission risk intervention strategies according to local conditions in different types of endemic areas, in order that further reduce the transmission risk of schistosomiasis and consolidate the achievements of schistosomiasis control.

Spatial autocorrelation, hot spots detection, density analysis and other methods in spatial epidemiology are commonly used to explore the distribution characteristics, transmission risk and influencing factors of infectious diseases. *O*. *hupensis* is the only intermediate host of *S*. *japonicum*, and is an important part in the transmission of schistosomiasis. The distribution of snails is closely related to the prevalence of schistosomiasis. The study on the spatial and temporal distribution pattern of snails can understand the distribution evolution of snails in each year, and detect the transmission risk of schistosomiasis timely, so as to realize early detection and control in key risk areas. In this study, global Moran’s *I* spatial autocorrelation analysis was conducted on the average density of living snails in Jiangling County from 2005 to 2021. The results showed that the average density of living snails in Jiangling County showed spatial autocorrelation in each year, that is, there were some spatial clustering areas in the average density of living snails in each year. The local spatial autocorrelation analysis showed the spatial correlation degree of each region in the average density of living snails in Jiangling County. The results showed that there was a certain mobility in the snails clustering area, which may be related to agricultural flood-drought rotation, flood disaster in flood season, and Yangtze River irrigation [43–45]. However, the clustering areas are mainly distributed around the west main ditch in the central part of Jiangling County and the eastern part of Jiangling County, among which some villages in Xionghe Town, Baimasi Town and Shagang Town have always been the clustering areas, which is consistent with the previous research results [7,8,46]. The reason may be that these villages are located near the west main ditch, which has a large water area and humidity conditions that may be more suitable for snail breeding and reproduction, leading to the easy rebound of snails epidemic situation. Hot spots analysis can detect the specific classification of hot spots areas on the basis of local spatial autocorrelation. The hot spot analysis results of this study are basically consistent with the “high-high” clustering areas detected by local spatial autocorrelation. The mean center and standard deviation ellipse analysis showed that the clustering areas of the average density of living snails in Jiangling County moved from northwest to southeast during 2005–2011, and then returned from southeast to northwest after 2014. The ellipse covered a wide area, but the annual variation was small, indicating that the area of snails was widely distributed, and the snails epidemic situation was prone to relapse, but the snails epidemic situation did not spread significantly. In this study, the risk level of schistosomiasis in Jiangling County was classified by kernal density analysis. The results showed that there was one high risk area of schistosomiasis in Jiangling County from 2005 to 2021, and the high and medium-high risk areas were concentrated in the southeast of Baimaisi Town, the east of Xionghe Town, the middle of Shagang Town and the north of Qinshi Town. It is consistent with the results of local autocorrelation analysis and hot spots analysis. The schistosomiasis transmission risk clustering areas detected by several methods in this study were all areas in Jiangling County where snails epidemic situation was more serious and easy to rebound.

Numerous studies have shown that the areas of emerging and re-emerging snails habitats have been detected during schistosomiasis surveillance in China in various years, posing potential risks for schistosomiasis transmission [47,48]. Since no schistosomiasis infected snails were detected in China in 2014, an area of 1.96hm^2^ was detected in China in 2020 [48,49]; The results of snails surveillance in national schistosomiasis surveillance sites from 2015 to 2019 showed that a total of 18 mixed snail samples with positive schistosomiasis nucleic acid were detected by loop-mediated isothermal amplification (LAMP) method, of which 83.33% were distributed in lake and marshland endemic area [50]; In 2021, schistosomiasis infected snails environments were detected in Hubei and Jiangxi provinces [47]. Therefore, although schistosomiasis is in a low epidemic state in China currently, the snails habitats area is widely distributed and the habitats of emerging and re-emerging snails are numerous. In the context of ecological protection policies such as the protection of the Yangtze River, environmental reconstruction projects and large-scale snails elimination with drugs are difficult to implement, and the risk of schistosomiasis transmission is still widespread [50]. Strengthening snails surveillance and control is the most important task for schistosomiasis control in the future. Therefore, it is recommended that Jiangling County continue to strengthen the surveillance and early warning of schistosomiasis transmission risk clustering areas, focusing on strengthening the monitoring of snails, expanding the monitoring scope and increasing the number of test samples, so as to timely detect the spread trend of snails. The monitoring frequency should be increased in the adjacent areas where snails are clustered and in areas without snails, so as to understand the diffusion risk of snails timely and prevent snails importation strictly. In addition, health education intervention should be strengthened in key areas. Currently, Jiangling County has reached the standard of schistosomiasis transmission interruption, and residents are easy to slack their awareness of schistosomiasis prevention. It is necessary to publicize the knowledge of schistosomiasis control regularly to raise the awareness of schistosomiasis prevention among residents and lay a solid foundation for achieving the goal of schistosomiasis elimination [51].

This study was subject to some limitations: First, the low sensitivity of the survey method may lead to the underestimation of schistosomiasis epidemic indicators in the stage of low prevalence of schistosomiasis, suggesting that the development of highly sensitive detection methods is urgent after transmission interruption. Second, this study was conducted on the scale of administrative villages, and future studies can be more detailed based on smaller scales. Third, this study analyzed the spatial and temporal clustering of schistosomiasis in Jiangling County and the transmission risk in different areas, but did not study the related factors affecting the clustering. Numerous studies have shown that the prevalence of schistosomiasis is to some extent influenced by natural factors such as climate and geographical environment, as well as factors such as socioeconomic level, suggesting that analysis can incorporate factors such as climate, geography and social economy in the future, in order to provide a basis for precise prevention and control of schistosomiasis [52,53].

## 5. Conclusions

This study analyzed the schistosomiasis transmission risk in different regions in Jiangling County, and targeted transmission risk intervention strategies can be adopted in different endemic areas after schistosomiasis transmission interruption. The schistosomiasis control in Jiangling County has achieved some effectiveness, but the snails distribution still showed spatial clustering in some areas. The areas with high risk of schistosomiasis transmission in Jiangling County were mainly distributed in the central part of Jiangling County near the west main ditch and the eastern part of Jiangling County. It is suggested strengthen schistosomiasis risk monitoring in Jiangling County, especially snails monitoring and snails elimination. In addition, health education intervention should be strengthened in key areas to prevent the spread of transmission risk to surrounding areas, in order to achieve the goal of eliminating schistosomiasis as soon as possible.
